# Supplementary material for: Pathway-Wide Genetic Risks in Chlamydial Infections Overlap between Tissue Tropisms: A Genome-Wide Association Scan
Source: Mediators Inflamm. 2018 Jun 3;2018:3434101. doi: 10.1155/2018/3434101 (PMC6008910; doi:10.1155/2018/3434101)
Supplement: Supplementary 2 — Supplementary Figure 1: results of GWAS analysis using EMMAX|QQ plot. Supplementary Table 1: complete list of SNPs with pEMMAX < 1 × 10^–6^. Supplementary Table 2: permuted p values for Reactome pathways with significant enrichment in chlamydial seropositivity: ALIGATOR analysis. [file 3434101.f2.docx]

**Supplementary Materials**

**Pathways-wide genetic risks in Chlamydial infections overlap between tissue tropisms : A genome-wide association scan.**

Chrissy h. Roberts^1#*^, Sander Ouberg^2#^, Mark Preston^3^, Martin J Holland^1^, Servaas Morre^2^

1. London School of Hygiene and Tropical Medicine, London, UK

2. Institute for Public Health Genomics, Maastricht University, The Netherlands

3. National Institute for Biological Standards and Controls, Potters Bar, UK

# : These authors contributed equally to the work

* Corresponding author.

Mailing address :

Clinical Research Department, London School of Hygiene and Tropical Medicine, Keppel St. London, UK WC1E 7HT

Phone : +44 (0) 20 7927 2913

E-mail : chrissy.roberts@lshtm.ac.uk


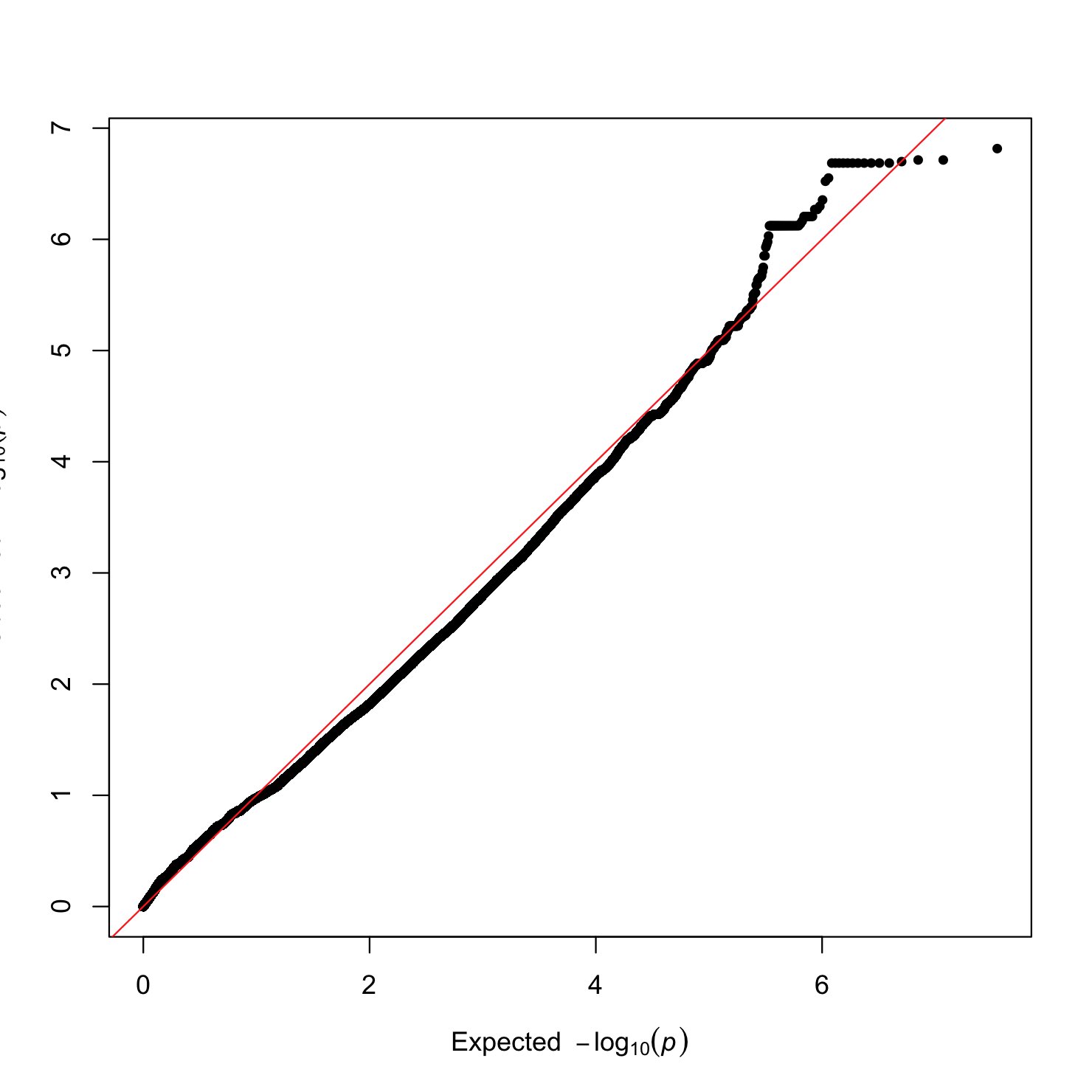


**Supplementary Figure 1: Results of GWAS Analysis using EMMAX | QQ plot**: There is negligible genome-wide inflation (λ = 1.02) of the test statistic when the phenotype is corrected for kinship using EMMAX.

**TABLES**

**Supplementary Table 1 : Complete list of SNPs with P_EMMAX_ < 1 x 10^-6^**

| SNP | CHR | BP | Allele1/2  (Effect allele bold) | | FREQ EA | Type | P | OR | Gene |
| --- | --- | --- | --- | --- | --- | --- | --- | --- | --- |
| rs6821248 | 4 | 104158360 | G | **A** | 0.253 | IMPUTED | 8.76E-07 | 0.79 | - |
| rs140848189 | 7 | 34560375 | G | **A** | 0.047 | 0 | 1.93E-07 | 0.59 | NPSR1/NPSR1-AS1 |
| rs76846649 | 7 | 34563394 | C | **T** | 0.047 | 0 | 2.06E-07 | 0.59 | NPSR1/NPSR1-AS1 |
| rs77039854 | 7 | 34568481 | T | **C** | 0.047 | 0 | 2.06E-07 | 0.59 | NPSR1/NPSR1-AS1 |
| rs80117932 | 7 | 34578732 | C | **T** | 0.047 | 0 | 2.06E-07 | 0.59 | NPSR1/NPSR1-AS1 |
| rs150504988 | 7 | 34579413 | C | **T** | 0.047 | 0 | 2.06E-07 | 0.59 | NPSR1/NPSR1-AS1 |
| rs144301570 | 7 | 34579601 | C | **T** | 0.047 | 0 | 2.06E-07 | 0.59 | NPSR1/NPSR1-AS1 |
| rs720756 | 7 | 34586240 | T | **C** | 0.047 | 0 | 2.06E-07 | 0.59 | NPSR1/NPSR1-AS1 |
| rs117759681 | 7 | 34587384 | A | **G** | 0.047 | 0 | 2.06E-07 | 0.59 | NPSR1/NPSR1-AS1 |
| rs117248409 | 7 | 34592104 | G | **C** | 0.047 | 0 | 2.06E-07 | 0.59 | NPSR1/NPSR1-AS1 |
| rs149816411 | 7 | 34603206 | G | **T** | 0.046 | 0 | 2.06E-07 | 0.59 | NPSR1/NPSR1-AS1 |
| rs117769079 | 7 | 34604834 | G | **A** | 0.046 | 0 | 2.06E-07 | 0.59 | NPSR1/NPSR1-AS1 |
| rs150769419 | 7 | 34616557 | C | **T** | 0.046 | 0 | 7.55E-07 | 0.61 | NPSR1/NPSR1-AS1 |
| rs143522867 | 7 | 34619964 | T | **C** | 0.047 | 0 | 6.86E-07 | 0.61 | NPSR1/NPSR1-AS1 |
| rs149492529 | 7 | 34622072 | C | **T** | 0.046 | 0 | 7.55E-07 | 0.61 | NPSR1/NPSR1-AS1 |
| rs78892190 | 7 | 34624324 | C | **T** | 0.05 | 0 | 1.53E-07 | 0.60 | NPSR1/NPSR1-AS1 |
| rs147718876 | 7 | 34624550 | A | **C** | 0.046 | 0 | 7.55E-07 | 0.61 | NPSR1/NPSR1-AS1 |
| rs10486647 | 7 | 34625366 | G | **A** | 0.046 | 0 | 7.55E-07 | 0.61 | NPSR1/NPSR1-AS1 |
| rs10486648 | 7 | 34626638 | G | **T** | 0.046 | 0 | 7.55E-07 | 0.61 | NPSR1/NPSR1-AS1 |
| rs117769298 | 7 | 34628434 | C | **T** | 0.046 | 0 | 7.55E-07 | 0.61 | NPSR1/NPSR1-AS1 |
| rs17185518 | 7 | 34629366 | C | **T** | 0.046 | 0 | 7.55E-07 | 0.61 | NPSR1/NPSR1-AS1 |
| rs144762542 | 7 | 34632200 | C | **T** | 0.046 | 0 | 7.55E-07 | 0.61 | NPSR1/NPSR1-AS1 |
| rs146348877 | 7 | 34634739 | T | **G** | 0.046 | 0 | 7.55E-07 | 0.61 | NPSR1/NPSR1-AS1 |
| rs149606913 | 7 | 34635012 | T | **C** | 0.046 | 0 | 7.55E-07 | 0.61 | NPSR1/NPSR1-AS1 |
| SNP | CHR | BP | Allele1/2  (Effect allele bold) | | FREQ EA | Type | P | OR | Gene |
| rs17766476 | 7 | 34635829 | A | **G** | 0.046 | 0 | 7.55E-07 | 0.61 | NPSR1/NPSR1-AS1 |
| rs143994032 | 7 | 34637735 | G | A | 0.046 | 0 | 7.55E-07 | 0.61 | NPSR1/NPSR1-AS1 |
| rs118100793 | 7 | 34638789 | C | T | 0.047 | 0 | 7.55E-07 | 0.61 | NPSR1/NPSR1-AS1 |
| rs17766746 | 7 | 34645369 | A | G | 0.046 | 0 | 7.55E-07 | 0.61 | NPSR1/NPSR1-AS1 |
| rs17186189 | 7 | 34648291 | T | C | 0.046 | 0 | 7.55E-07 | 0.61 | NPSR1/NPSR1-AS1 |
| rs10259060 | 7 | 34656439 | T | C | 0.046 | 0 | 7.55E-07 | 0.61 | NPSR1/NPSR1-AS1 |
| rs10257538 | 7 | 34661232 | T | G | 0.044 | 0 | 2.00E-07 | 0.59 | NPSR1/NPSR1-AS1 |
| rs10270133 | 7 | 34664232 | T | C | 0.046 | 0 | 7.55E-07 | 0.61 | NPSR1/NPSR1-AS1 |
| rs28615557 | 7 | 34666825 | T | C | 0.046 | 0 | 7.55E-07 | 0.61 | NPSR1/NPSR1-AS1 |
| rs10244727 | 7 | 34666861 | G | T | 0.046 | 0 | 7.55E-07 | 0.61 | NPSR1/NPSR1-AS1 |
| rs2169679 | 10 | 53873323 | C | T | 0.326 | 0 | 9.33E-07 | 0.81 | PRKG1 |
| rs10762570 | 10 | 53878170 | C | T | 0.324 | 0 | 5.04E-07 | 0.80 | PRKG1 |
| rs12259288 | 10 | 53889255 | A | G | 0.333 | 0 | 2.81E-07 | 0.8 | PRKG1 |
| rs4082299 | 10 | 53896068 | T | C | 0.322 | 0 | 5.39E-07 | 0.81 | PRKG1 |
| rs12413303 | 10 | 53896150 | G | A | 0.322 | 2 | 6.23E-07 | 0.81 | PRKG1 |
| rs10824089 | 10 | 53896170 | G | C | 0.322 | 0 | 6.23E-07 | 0.81 | PRKG1 |
| rs4935310 | 10 | 53896500 | A | G | 0.322 | 0 | 3.01E-07 | 0.80 | PRKG1 |
| rs4935032 | 10 | 53896713 | T | C | 0.322 | 0 | 6.23E-07 | 0.81 | PRKG1 |
| rs4935311 | 10 | 53896741 | G | T | 0.322 | 0 | 6.23E-07 | 0.81 | PRKG1 |
| rs10762578 | 10 | 53897291 | C | G | 0.323 | 0 | 7.25E-07 | 0.81 | PRKG1 |
| rs10762580 | 10 | 53897482 | C | T | 0.322 | 0 | 6.23E-07 | 0.81 | PRKG1 |
| rs3886884 | 10 | 53898181 | A | G | 0.322 | 0 | 5.39E-07 | 0.81 | PRKG1 |
| rs77175455 | 16 | 29615810 | **A** | T | 0.62 | IMPUTED | 7.43E-07 | 0.75 | *-* |
| rs79741827 | 16 | 56733116 | T | C | 0.044 | 0 | 4.42E-07 | 0.59 | - |

**Supplementary Table 2 : Permuted P values for Reactome pathways with significant enrichment in Chlamydial seropositivity: ALIGATOR analysis.**

|  | Cutoff threshold | P = 0.01 | | P = 0.001 | P = 0.0001 |
| --- | --- | --- | --- | --- | --- |
| Pathway Name |  | P values for tests | | | |
| GPCR downstream signaling | | | 0.00009 | 0.349 | 0.5294 |
| Olfactory Signaling Pathway | | 0.00009 | | 0.2862 | 0.6036 |
| Signaling by GPCR | | 0.0006 | | 0.2604 | 0.3868 |
| Signal Transduction | | 0.0014 | | 0.2554 | 0.2146 |
| G1 S Transition | | 0.0052 | | 0.8932 | 0.2158 |
| CREB phosphorylation through the activation of CaMKII | | 0.007 | | 1 | 1 |
| Mitotic G1 G1 S phases | | 0.0168 | | 0.7666 | 0.0382 |
| CRMPs in Sema3A signaling | | 0.0206 | | 0.2692 | 1 |
| The citric acid TCA cycle and respiratory electron transport | | 0.0334 | | 0.0036 | 1 |
| P2Y receptors | | 0.0352 | | 1 | 1 |
| Regulation of DNA replication | | 0.039 | | 1 | 1 |
| Removal of licensing factors from origins | | 0.039 | | 1 | 1 |
| Sphingolipid de novo biosynthesis | | 0.0394 | | 0.8668 | 0.211 |
| Cyclin E associated events during G1 S transition | | 0.0428 | | 0.7174 | 0.134 |
| p53 Dependent G1 DNA Damage Response | | 0.0478 | | 0.6754 | 0.1164 |
| p53 Dependent G1 S DNA damage checkpoint | | 0.0478 | | 0.6754 | 0.1164 |
| G alpha i signalling events | | 0.0484 | | 0.0342 | 1 |
| Cyclin A B1 associated events during G2 M transition | | 0.0616 | | 0.3126 | 0.0408 |
| Retinoid cycle disease events | | 0.0792 | | 0.1332 | 0.0166 |
| Respiratory electron transport, ATP synthesis by chemiosmotic coupling, and heat production by uncoupling proteins. | | 0.0882 | | 0.0054 | 1 |
| Proton oligonucleotide cotransporters | | 0.093 | | 0.0122 | 1 |
| Eicosanoids | | 0.105 | | 0.0618 | 0.0448 |
| AKT phosphorylates targets in the cytosol | | 0.16 | | 0.0372 | 0.032 |
| Chemokine receptors bind chemokines | | 0.1936 | | 0.0432 | 1 |
| Constitutive PI3K AKT Signaling in Cancer | | 0.1954 | | 0.076 | 0.0336 |
| Respiratory electron transport | | 0.2006 | | 0.0388 | 1 |
| Synthesis of PA | | 0.2034 | | 0.0498 | 1 |
| FGFR2b ligand binding and activation | | 0.2356 | | 0.0248 | 0.0366 |
| Mitochondrial Iron Sulfur Cluster Biogenesis | | 0.2366 | | 0.1944 | 0.0262 |
| RNA Polymerase I Promoter Opening | | 0.3196 | | 0.0412 | 1 |
| Citric acid cycle TCA cycle | | 0.3198 | | 0.0476 | 1 |
| FGFR2c ligand binding and activation | | 0.3362 | | 0.264 | 0.0364 |
| Growth hormone receptor signaling | | 0.3568 | | 0.0204 | 1 |
| Binding and entry of HIV virion | | 0.3934 | | 0.0468 | 1 |
| The canonical retinoid cycle in rods twilight vision | | 0.405 | | 0.2652 | 0.028 |
| Insulin effects increased synthesis of Xylulose 5 Phosphate | | 0.4224 | | 0.0392 | 1 |
| Ion transport by P type ATPases | | 0.4334 | | 0.257 | 0.0382 |
| PI3K AKT activation | | 0.4702 | | 0.1434 | 0.0486 |
| PI 3K cascade | | 0.4702 | | 0.1434 | 0.0486 |
| PI3K events in ERBB2 signaling | | 0.4702 | | 0.1434 | 0.0486 |
| PI3K events in ERBB4 signaling | | 0.4702 | | 0.1434 | 0.0486 |
| PI3K AKT Signaling in Cancer | | 0.4702 | | 0.1434 | 0.0486 |
| PIP3 activates AKT signaling | | 0.4702 | | 0.1434 | 0.0486 |
| Rho GTPase cycle | | 0.4972 | | 0.4676 | 0.029 |
| Signaling by Rho GTPases | | 0.4972 | | 0.4676 | 0.029 |
| Endosomal Sorting Complex Required For Transport ESCRT | | 0.505 | | 0.0254 | 1 |
| RNA Polymerase I Chain Elongation | | 0.5396 | | 0.0604 | 0.0384 |
| RNA Polymerase I Transcription Initiation | | 0.5852 | | 0.0702 | 0.0414 |
| RNA Polymerase I Promoter Escape | | 0.5852 | | 0.0702 | 0.0414 |
| PD 1 signaling | | 0.592 | | 0.034 | 1 |
| Cell Cycle | | 0.603 | | 0.4332 | 0.038 |
| RNA Polymerase I Promoter Clearance | | 0.6164 | | 0.0756 | 0.0428 |
| RNA Polymerase I Transcription Termination | | 0.6256 | | 0.0748 | 0.0422 |
| Dual incision reaction in GG NER | | 0.6366 | | 0.3946 | 0.0438 |
| Formation of incision complex in GG NER | | 0.6366 | | 0.3946 | 0.0438 |
| Signaling by VEGF | | 0.6518 | | 0.0344 | 1 |
| VEGF ligand receptor interactions | | 0.6518 | | 0.0344 | 1 |
| Signaling by FGFR2 amplification mutants | | 0.653 | | 0.1074 | 0.0144 |
| Cytochrome P450 arranged by substrate type | | 0.6536 | | 0.0412 | 0.1708 |
| RNA Polymerase I Transcription | | 0.6904 | | 0.0904 | 0.0466 |
| Free fatty acid receptors | | 0.7174 | | 0.1326 | 0.0164 |
| Mitochondrial tRNA aminoacylation | | 0.7246 | | 0.0452 | 1 |
| Type I hemidesmosome assembly | | 0.742 | | 0.2836 | 0.0348 |
| Tandem pore domain halothane inhibited K+ channel THIK | | 0.8106 | | 0.139 | 0.0214 |
| Neurotransmitter uptake and Metabolism In Glial Cells | | 0.843 | | 0.2284 | 0.0242 |
| Astrocytic Glutamate Glutamine Uptake And Metabolism | | 0.843 | | 0.2284 | 0.0242 |
